# ASMR as Idiosyncratic Experience: Experimental Evidence

**DOI:** 10.3390/ijerph182111459

**Published:** 2021-10-30

**Authors:** Chiara Pedrini, Lorena Marotta, Andrea Guazzini

**Affiliations:** 1 Department of Education, Languages, Intercultures, Literatures and Psychology, University of Florence, via di San Salvi, 12, Building 26, 50100 Florence, Italy; lorenamarotta11@gmail.com (L.M.); andrea.guazzini@unifi.it (A.G.); 2 Center for Study of Complex Dynamics (CSDC), University of Florence, 50100 Florence, Italy

**Keywords:** ASMR, pupil diameter, NeuroSky brain waves, arousal, idiosyncratic experiences

## Abstract

The Autonomous Sensory Meridian Response (ASMR) is a tingling sensation across the scalp that occur in response to specific triggering audio and visual stimuli, connected with the Default Mode Network. Our study (N = 76) aimed to test the neurophysiology of ASMR by examining pupil diameter and brain activity. Assuming the idiosyncratic nature of ASMR, we expected results detecting opposite physiological outcomes considering pupil diameter and brain activation. We used a battery of self-reports to investigate psychological dimensions; for the physiological measures, we used two instruments: PupilCore and NeuroSky MindWave Mobile 2. The results showed an augmented pupillary diameter during the ASMR video, regardless of the perception of tingles. On the other hand, the arousal level during the ASMR video was lower than the other conditions. The difference between the two neurophysiological measures appeared as peculiar and can be considered as the promoting phenomenon for ASMR psychological outcomes.

## 1. Introduction

The autonomous sensory meridian response (ASMR) is a sensory phenomenon typically characterized by tingling across the scalp, following the line of the spine downward, extending to the arms and further depending on the intensity of the response [1]. Assuming a complex idiosyncratic reaction in the sample, with this study, we examined the ASMR experience by observing the pupillary diameter and the arousal level. Our findings showed differences in pupillary diameter, regardless of the perception of tingles, and in the arousal level, in comparison with other video conditions. Furthermore, we utilized self-report measures to explore the ASMR condition, finding significant differences in the Telepresence Scale and Interpersonal Reactivity Index Scale. ASMR is a complex phenomenon mediated by the viewing of videos that are proven to induce tingling sensations across the scalp, following the line of the spine downwards, extending to the arms and further depending on the intensity of the response [1]. Tingles are elicited by a number of audio and visual triggers—such as whispers, crispy sounds, personal care, and slow or repetitive movements. The experience of ASMR “tingles” is often accompanied by feelings of calm and relaxation [2,3], which the subjects compare to falling asleep. ASMR was recently brought to the attention of the public and a few examples of research have been published about the phenomenon since 2015. Some of these have analyzed the relation that ASMR has with depression and anxiety [1], intimacy [4], emotional regulation strategies [5], personality traits and empathy [6], telepresence [7], and other psychological aspects. The underlying mechanisms in ASMR are still understudied. In a study examining the default mode network (DMN) in relation to the ASMR, Smith et al. [8,9] using fMRI found that ASMR experiencers reported reduced resting-state functional connectivity between frontal, sensory, and attentional regions of the DMN. Lochte et al. [10] using fMRI found that ASMR experiencers showed significant activation in the medial Prefrontal Cortex (mPFC) associated with self-awareness and social cognition, in the Nucleo Accumbens (NAcc) associated with reward, and in the Anterior Cingulate Cortex (dACC) and Insula associated with emotional arousal. Furthermore, Wang et al. [11] investigated the influence of ASMR on the performance of executive functions in both ASMR and control individuals, finding that some executive functions of ASMR individuals slowed down after the ASMR video (specifically set shifting and inhibitory control). Finally, recent studies compared brain activity studied through the use of EEG: Fredborg et al. [12] examined brain waves related to ASMR through the use of electroencephalography (EEG). They found that ASMR auditory stimuli increased alpha wave activity (alpha power—in addition to theta—has been associated with meditative states [13]) in participants with self-reported ASMR. This did not seem to occur in control participants. Seifzadeh et al. [14] examined the variations in EEG frequency bands in pre-ASMR versus post-ASMR [14]. Finally, Koo et al. [15] found that arousal was a key feature of ASMR [15]. In 2018, Poerio et al. [2] compared ASMR tingles with the well-known aesthetic chills recognizing similarities between the two phenomena; considering both as phenomenologically complex and idiosyncratic experiences, they asserted that ASMR is typically considered to be relaxing, while aesthetic chills are associated with excitement and physiological arousal. The findings of the study showed that ASMR is a relaxing experience also accompanied by arousing effects, such as increased skin conductance levels. In line with recent literature that adopted eye-tracking and brain waves to study various fields of application, including cognition [16], arousal [17], marketing [18], and, more recently, advanced learning technologies [19], we conducted experimental research in which we combined such technologies to investigate arousal level under ASMR conditions.

Regarding eye tracking, the first study about pupillometry and ASMR was conducted by Valtakari et al. [20]; they found out that ASMR videos cause a statistically significant increase in pupil diameter (4.07 mm), observing a significant difference only during reported episodes of tingles.

Several arousal studies used pupillary diameter measurement [21,22], finding that, in general, the pupil tends to dilate in subjects submitted to attentional stimuli, triggering an elevation in the arousal (the arousal in these studies was further measured by detecting the heartbeat or skin conductance). In this way, pupil size proved to be a reliable indicator of emotional arousal, and it is also related to other physiological arousal measures, such as skin conductance [21]. Furthermore, as discussed by Steinhauer et al. [23], changes in pupil diameter are controlled by two muscles—the dilator and the sphincter—which are differentially influenced by activity in the sympathetic and parasympathetic systems. Increased sympathetic activity amplifies the activity of the dilator muscle, causing dilation, whereas activity of the parasympathetic system causes the constriction of the sphincter muscle, which results in a decrease in pupil diameter. Taking these argumentations as a point of departure, we assumed that ASMR experience could be characterized by activating as much as deactivating responses, on a physiological and psychological level. In order to verify this assumption, the aim of the study was the measurement of the ASMR phenomenon, through the use of two tools. We compared an ASMR video with other contents—resting state, high arousal, low arousal, control videos—and, at the same time, we submitted a battery of self-reported tests, regarding psychological variables and different aspects of the ASMR experience. About the tools, we decided to use two new instruments for the detection of physiological measures: NeuroSky MindWave Mobile 2, connectable to a laptop via Bluetooth, allows one to collect data around brain frequencies, states of attention and relaxation, and also blinks; PupilCore, a monocular or binocular eye tracking device developed by Pupil Labs, includes an open-source software suite and a wearable headset. We expected that, during the viewing of an ASMR video, the participants would show a greater average pupil diameter increase compared to other video contents. Furthermore, we expected, under the same condition, an increased brain activity, also confirmed by results in the self-report measures.

**Hypothesis 1** **(H1).** *People during the viewing of an ASMR video will have a larger pupil diameter compared to the viewing of other videos.*

**Hypothesis 2** **(H2).** *People during the viewing of an ASMR video will have a general reduction in arousal, measured by Neurosky brain waves, compared to the viewing of other videos.*

**Hypothesis 3** **(H3).** *ASMR experiencers will have different scores in psychological dimensions compared to Non-ASMR experiencers.*

**Hypothesis 4** **(H4).** *People with high values in ASMR-15 will have a higher brain activation.*

## 2. Method and Procedures

### 2.1. Sample

A total of 76 participants were recruited for the experiment (females = 53, 69, 7%; males = 23, 30, 3%). Participants were invited to take part in the experiment with e-mails and private messages. The sample was composed by young adults (*M* = 24.96 *SD* = 4.867). The participation was completely voluntary, and anyone who wanted to be involved as an experimental subject had to sign an informed consent statement.

### 2.2. Stimuli

#### 2.2.1. Physiological Measures

NEUROSKY MINDWAVE MOBILE 2: this device is a wireless electroencephalogram that allows one to trace an EEG in which different brain waves (Alpha, Beta, Gamma, Delta, and Theta), the number of blinks, and three composite measures called “*Attention,*” “*Meditation,*” and “*Zones*” can be clearly delineated. As stated on the website [24]: “MindWave Mobile 2 EEG headsets are the culmination of decades of EEG biosensor technology research—all in one easy-to-control, wearable package.” The device is very simple and slight: it consists of an earphone, an ear clip, and a sensor holder. The two electrodes are located on the ear clip (left) and on the left of the forehead (frontal area). Through the MindPlayer software, to which the NeuroSky device is connected via Bluetooth, it is possible to start recording and export an output of the final track [25]. The EEG values are expressed typically in Volts-squared per Hz (V^2^/Hz), but the NeuroSky Agency has chosen to refer to the unit of measure as ASIC-EEG-POWER units, as you can read on the website [25]. The choice of this device was due to the fact that it appeared to be portable and affordable, and not very sensitive to motion artifacts. Nevertheless, it is substantially more usable for evaluating general arousal than for the accuracy of the individual frequencies [26,27].

PUPILCORE: The PupilCore is a monocular eye-tracking device from Pupil Labs, featuring an open-source software suite and a wearable headset [28].

It has two cameras, one tracking the subject’s pupil and one framing their vision of the ‘world’; the support is light and adjustable, based on the characteristics of the user’s face. Both cameras connect to a laptop platform via USB. The camera outputs are read through Pupil Capture software for real-time pupil detection, gaze mapping, recording, and other functions. The data acquisition program (Pupil Player) has an interface that presents various options for customizing the recording, as well as calibrating the subject’s field of vision.

#### 2.2.2. Videos

We selected as stimuli for the present study 5 videos with different contents. The ASMR video and the Control video with a duration of 30 s were selected from another study about the ASMR phenomenon [29]. Videos of High Arousal and Low Arousal were selected from a public database from the University of Stanford [30] with a duration of 30 s; for the High-Arousal video, we chose the “Canyon Swing” video (Valence 5.38; Arousal 6.88), while for the Low Arousal video, we chose the “Blyde Canyon” video (Valence 4.82; Arousal 3.09). Lastly, the REST video was specifically created by the researchers. It was a 30 s video showing a white screen with a fixed black dot in the center. The participants were asked to keep their gaze fixed on the dot for the duration of the video. A fixed dot was chosen to induce a “resting state,” as suggested by studies of Owens and Leibowitz [31] and Thaler et al. [32]. In these studies, it was shown how the vision of a fixed dot for a certain amount of time induces a sort of resting state.

We chose ASMR and Control videos from Cash et al. [29] research because, in this study, it was demonstrated that these videos could (or could not) induce ASMR phenomena.

Likewise, we chose High-Arousal and Low-Arousal videos from Li et al.’s [30] database because it is demonstrated that these videos could induce different levels of Arousal. Lastly, the REST video was used principally to compare a resting state condition with other videos; furthermore, it was used as an intermission between one phase and another, and to induce a baseline condition in subjects before and after a detection phase.

#### 2.2.3. Self-Report Measures

The procedure of the experiment included filling out different questionnaires in order to investigate various psychological aspects of the sample.

*Socio-demographic survey*, which included age and gender.

*Interpersonal Reactivity Index (IRI)* [33]: a test used for the dimensional assessment of empathy, consisting of 28 items on a 5-point Likert scale. The test has 4 subscales, each consisting of 7 different items. These subscales are: Perspective-Taking Scale—the tendency to spontaneously adopt the psychological point of view of others; Fantasy Scale—it draws on the tendencies of transpose themselves imaginatively into the feelings and actions of fictitious characters in books, films, and plays; Empathic Concern Scale—evaluates “other-oriented” feelings of sympathy and concern for the unfortunate; Personal Distress Scale—evaluates “self-oriented” feelings of personal anxiety and discomfort in interpersonal contexts (Cronbach’s ***α***: PT = 0.75, EC = 0.80, PD = 0.76, FS = 0.79).

*Telepresence Scale* [34]: a self-report scale that involves 8 items on a Likert scale from 1 (Totally Disagree) to 7 (Totally agree), investigating the experience of telepresence lived by the subject (Cronbach’s ***α*** 0.78). Telepresence is defined as a person’s perception of being in a specified or understood virtual place [34]. Even if there are no virtual environments, the degree of immersion elicited by the video experience is a standard usually assessed with this scale.

To investigate the ASMR phenomenon, we chose 3 different surveys submitted post-video session:

*ASMR Experience* [2]: a single-item interview about the subject’s ASMR experience. The question asked to the subject was how much the subject had experienced ASMR—“How frequently (if at all) did you experience tingling sensations during the video?” from 1 (none of the time) to 7 (all of the time).

*ASMR-15* [35]: a questionnaire of 15 items concerning the proven ASMR experience. The 4 scales in which the questionnaire is divided concern: AC—Altered Consciousness (4 items); S—Sensation (5 items); R—Relaxation (3 items); A—Affect (3 items). The answers were expressed on a Likert scale from 1 (totally false for me) to 5 (totally true for me) (Cronbach’s ***α*** 0.78).

*ASMR POST* [29]: an interview consisting of 12 items that investigates the status of ASMR perceived by the subjects, through qualitative and quantitative questions.

### 2.3. Experimental Procedures

The experiment took place in a laboratory of the Department of Psychology of the University of Florence. The procedure was divided into 3 experimental phases, and included PRE-self-report administration, eye-tracking, and Neurosky brain waves detection for the duration of each video, and POST-Self report administration. The order of the video presentation was balanced to eliminate possible priming effects; in particular, we alternated the order of the ASMR video and CONTROL video, as well as the HIGH- and LOW-AROUSAL video. Each video was separated by the viewing of a REST video. The setting was arranged to promote the isolation of the subject: the person was alone in a quiet room with the computer used to follow the procedure, wore earphones, and was assigned an experimental ID to keep the participants anonymous. We registered a 6% sampling mortality rate between acceptance to participate and real participation. The criteria for inclusion in this study were: to have the ability to give consent in the experiment participation, to have the ability to correctly understand and fluently speak Italian, and to be able to watch a computer display at a distance of 54 cm without the use of glasses.

All distracting elements were removed from the room. The setting was maintained quiet for the entire duration of the experimental session. The setting of the experiment was standardized; in particular, the distance of the subject from the PC, the audio of the device, and the luminance were controlled: the luminance of the room was 50 lux, the computer screen brightness was set to 100%; the computer audio was set to 80%; the distance of the subject from the screen was 54 cm. There were two computers connected by a Google ChromeCast, one out of the room for the researchers, where they could follow the subjects filling out the report, and one in the room for the subjects, where the experimental procedure took place.

All the videos, and consequently the vectors considered to compute the average brain and pupil activity, had an identical duration (i.e., vector length) of 30 s. The researchers monitored in real time the subject through the use of internal cameras, and the Google ChromeCast, leaving the subject in a condition of physical isolation. The researchers reported the absolute time of beginning and end of any phases of the “trial” as triggers to be compared subsequently with the synchronized absolute time detailing the PupilCore and NeuroSky data stream. Finally, we decided to not renormalize the signals on their baseline conditions, but to consider such baselines in the data analysis as reference conditions. The experimental sessions always had a total duration of 30 min.

### 2.4. Data Analysis

As the first step of the analysis, we codified and cleaned the datasets coming from the NeuroSky and the PupilCore. The signal preprocessing was composed by three steps: (i) we cut the 30′′ temporal windows (i.e., experimental phases) for all the signals according to the experimental protocol; (ii) we cleaned the technological artefacts, removing 5% of extreme values (i.e., trimmed mean); (iii) we substituted the missing value (i.e., <5%) with a regression method interpolating the signal with the weighted moving average method (WMA). As the second step, analyses of frequencies, central tendencies, and dispersion indicators were carried out using the IBM Statistical Package for Social Sciences (SPSS 27.0) [36]. Before proceeding with inferential analyses, we checked that the data respected the analyses’ assumptions and, thus, we verified the normality of distribution of the continuous variables considered by the study, as well as the numerosity and balancing requirements. Given the structure of the data, we adopted linear correlation and Student’s *t* statistics to explore the experimental hypotheses.

## 3. Results

First, we cover the sociodemographic and descriptive outcomes. Secondly, we show the inferential statistics starting with the comparison between pupil diameter and brain waves frequencies among different video contents. Finally, we discuss the outcomes.

### 3.1. Descriptive Statistics

A total of 76 participants (69.7% females; 30.3% males) were recruited for the study. The sociodemographic information included age (*M* = 24.96, *SD* = 4.867) and gender (male, female). No statistically significant difference can be found with regard to sex and age.

### 3.2. Psychological Characteristics

We investigated the following psychological measures: empathy and telepresence. Specifically, the data were configured as follows (see Table 1):

*Empathy*: for the experiment, we used the Interpersonal Reactivity Index (IRI) [33].

The test has four subscales: Perspective-Taking Scale (*M* = 27, *SD* = 4.7), Fantasy Scale (*M* = 26.9, *SD* = 4.6), Empathic-Concern Scale (*M* = 27.1, *SD* = 4.5), Personal Distress Scale (*M* = 19.1, *SD* = 5.4).

*Telepresence*: for the experiment, we used the Telepresence Scale [34] after the ASMR experience. The test has two dimensions: Being present in the mediated environment, and Not being present in the mediated environment. For the second administration, the results were: Being present in the mediated environment (*M* = 18.5, *SD* = 5.0), Not being present in the mediated environment (*M* = 10.1, *SD* = 3.1).

### 3.3. Neurophysiological Characteristics

We investigated pupil diameter and brain waves for each video that was submitted to the sample. For pupil diameter, we report in Table 2 the mean diameter, *SD*, skewness, and kurtosis. ASMR determined the higher pupil diameter (*M* = 4.32, *SD* = 1.31) compared to the other videos (Rest, High Arousal, Low Arousal, Control). The data obtained from the differences between the conditions of Low Arousal, High Arousal, and Control videos were not significant.

For frequency bands results, we report in Table 3 the values (expressed in ASIC-EEG-POWER) of mean, *SD*, skewness, and kurtosis of the ASMR video. As the EEG variables did not distribute normally, they were log-transformed before correlation calculation. mean, skewness, and kurtosis refer to the 30 s frequency band measurement time window of each video. Even if it is not a standard way of presenting data, it was sufficient for the level of detail we were interested in.

### 3.4. Descriptive ASMR Experience

For the measurement of ASMR experience, we used ASMR-Experience [2] and ASMR 15 [33]. ASMR POST [29] administration did not produce significant results.

*ASMR-Experience* is composed of the item “How frequently did you experience tingling sensations during the video?”, rated on a Likert scale from 1 (none of the time) to 7 (all of the time). The results showed a mean of 1.97 (*SD* = 1.29).

*ASMR 15* has four subscales: Altered Consciousness (*M* = 8.1, *SD* = 4), Sensation (*M* = 11.6, *SD* = 5.6), Relaxation (*M* = 7.6, *SD* = 3.5), Affect (*M* = 7.4, *SD* = 3.4). Results are reported in Table 4.

### 3.5. Inferential Statistics

**H1: Pupil dilation during ASMR video compared to other conditions.**Table 5 illustrates the comparison between average pupil diameter for ASMR videos [29] and for other videos, showing that pupil diameter is always greater during ASMR videos. As a reminder, the other videos had different contents. In particular, the REST video showed a white screen with a fixed black dot in the center, to induce a “resting state,” as suggested by Owens and Leibowitz [31]; High-Arousal and Low-Arousal videos were selected from the database of Li et al. [30], to induce different levels of arousal; the Control video was selected from Cash et al.’s [29] research. The difference registered in pupil diameter with the ASMR and REST video was 0.92 (mm); therefore, this was the largest. On the contrary, the smallest difference in pupil diameter was registered in the ASMR and CONTROL video (only 0.36 mm). Of the conditions ASMR-HIGH AROUSAL and ASMR-LOW AROUSAL, the pupil diameter differed, respectively, by 0.53 mm and 0.43 mm.

**H2: Brain activation between ASMR and other contents.**Table 6 presents the results of a Student’s *t*-test for the EEG delta between the ASMR video and other videos. Delta values during the ASMR video were lower than during the High-Arousal (*t* = *−*2.12, *p <* 0.05), Low-Arousal (*t* = *−*2.61, *p <* 0.01), and Control videos (*t* = *−*2.94, *p <* 0.001). Theta values during the ASMR video were lower than during the Low-Arousal (*t* = *−*2.15, *p <* 0.05) and Control videos (*t* = *−*2.66, *p <* 0.01). Low Alpha differed only during the Low-Arousal video experience, being higher than during the ASMR video (*t* = *−*1.92, *p <* 0.05). High Alpha Delta appeared lower during the ASMR video than during the Low-Arousal (*t* = *−*3.49, *p <* 0.001) and Control videos (*t* = *−*2.56, *p <* 0.05). Low Beta values were lower during the ASMR video than during the High-Arousal, Low-Arousal, and Control videos, respectively, with *t* = *−*2.64, *p <* 0.01; *t* = *−*2.38, *p <* 0.05; and *t* = 2.02, *p <* 0.05. High Beta values did not present significant differences between the conditions, while Low Gamma (*t* = *−*2.89, *p <* 0.001) and Mid Gamma values (*t* = *−*2.19, *p <* 0.05) during ASMR were lower than during the Control video. Interestingly, from the brain activity point of view, the ASMR video did not show any differences with the Rest video.

**H3: Psychological measures in ASMR experiencers and Non-ASMR experiencers.** We found differences only in the correlation between brain wave dimensions; to investigate the predictors of ASMR experience (e.g., tingles perception), we took into account as a dependent variable the answers provided to the question “How frequently did you experience tingling sensations during the video?” Due to the non-Gaussian distribution of the answers, we first eliminated those participants who answered “never” (*n* = 39), in order to investigate only those participants who reported an ASMR experience to any degree. Further, in order to respect the inferential analysis preconditions, we discretized the variable within two levels, defining two balanced subsamples, respectively, labeled as “Few” and “Many” tingles perceived. The “Few” subsample was composed by those participants that reported tingles only a “Few times” (*n* = 17), while the “Many” subsample was composed by those who reported tingles “Sometimes” (*n* = 10), “Half the time” (*n* = 4), “Quite frequently” (*n* = 5), and “Frequently” (*n* = 1).

The differences between the subsample reporting more tingles experiences (“Many”) and the subsample that reported few tingles (“Few”) are summarized in Table 7; in particular, we present the results of a Student’s *t* test. Regarding IRI, higher scores in the subscale Empathic Concern appeared to determine higher tingles perception (*t* = 2.34, *p <* 0.05), but they reported lower scores in the subscale Personal Distress (*t* = *−*2.27, *p <* 0.05). The subscales “Perspective-Taking” and “Fantasy” were not statistically significant.

Of the Telepresence Scale, lower scores in the subscale “Being Present in the Mediated Environment” appeared to be associated with higher tingles receivers (*t* = *−*2.03, *p <* 0.05).

**H4: Higher brain activation with tingles experiences is shown by the ASMR-15 results.**Table 8 illustrates the correlations between frequency bands and ASMR-15 subscales scores. This analysis aimed to highlight any linear relationships between ASMR-15 subscales (as indicators of subjective psychological experience) and NeuroSky as an indicator of physiological arousal. The scores provided by the ASMR-15 subscale “Sensations” appeared to be related to almost all the frequency bands considered by our study, with the only exceptions of Low Alpha and High Alpha. The degree of sensation related to ASMR appeared to increase whenever the brain arousal increased. While the “Relaxation” scale scores did not show any significant correlation with brain arousal, the “Altered Consciousness” and “Affect” scores reported small correlations with Gamma (Altered consciousness and Affect) and Beta (Affect) waves.

## 4. Conclusions

Autonomous Sensory Meridian Response (ASMR) is a phenomenon still to be explored, but a large amount of research has analyzed its characteristics. For our study, to perform a multi-dimensional analysis, we chose to investigate ASMR through an eye-tracking device (PupilCore) and a wireless electroencephalogram (NeuroSky), concurrently with the submission of some self-report measures (IRI, Telepresence Scales, ASMR-Experience, and ASMR 15). The sensitivity to the phenomenon could be linked to different psychological characteristics, including greater sensory suggestibility [38].

For our study, we chose to investigate the influence of empathy levels through the Interpersonal Reactivity Index (IRI) [33], previously used also by McErlean and Banissy [6]. For the IRI-Empathic Concern subscale, we found that the subsample that felt more tingles (“Many”) reported a higher score that the other subsample (“Few”), according to McErlean and Banissy [6]; further, for the IRI-Personal Distress subscale, the subsample “Few” reported a higher score that the other subsample “Many.” In our opinion, according to Bjelić [39], ASMR could concern a sort of digital care, a kind of care transmitted through digital technology. According to the author, “The popularity of ASMR videos can be viewed as symptomatic of an existing social need to be cared for, loved, and connected with. Moreover, this kind of care is being sought through digital technology, through digital intimacy” [39]. In the same way, individuals with a higher IRI-Empathic Concern score—which evaluates “other-oriented” feelings of sympathy and concern for the unfortunate—could be more receptive toward the tingling phenomenon. Even Lochte et al. [10] claimed that ASMR could activate brain regions and release substances normally associated with affiliated behaviors (crinkling, tapping, brushing, or massaging). We also used the Telepresence Scale [34] to analyze the sense of presence within virtual environments. Comparing the two subsamples of lower vs. higher tingles receivers (“Few” and “Many”), we found that the subsample “Few” reported a higher score in the subscale Being Present in the Mediated Environment. Furthermore, we analyzed the ASMR sensations through two specific self-report measures: ASMR-Experience [2] and ASMR 15 [35]. By the comparison between the results of ASMR 15 and NeuroSky dimensions, our results showed different correlations between brain waves and the subscale Sensations (the subscale that explores the localization and the intensity of tingles). For this reason, we assumed that changes in brain activity were due to the perception of tingles.

Neurophysiological parameters are also involved in the ASMR phenomenon: for example, Poerio et al. [2] claimed that ASMR videos produced sensations of both calmness and excitement, measured by different neurophysiological tools; Lochte et al. [10] claimed that ASMR increased activity in brain regions typically related to reward activation, emotional arousal, and social engagement, suggesting that ASMR may be similar to social grooming; Fredborg et al. [12] reported an increased alpha, gamma, and sensorimotor rhythm activity, which may indicate changes in attentional control; in addition, Smith et al. [8,9] sustained that ASMR participants showed significantly reduced functional connectivity in the DMN brain regions. An important contribution to our study is given by Valtakari et al. [20]: they examined the tingles’ experiences and found them to be accompanied by a growing pupil diameter (4.03 mm), considering this as a physiological characteristic trait of ASMR-experiencers. Unexpectedly, our results reported that the increase in pupil diameter (4.32 mm) was present in the whole sample, regardless of the tingles experience. As much as these results need further insight, it seems that the whole experience of ASMR might involve different features, and it manifests itself in each subject, regardless of their sensitivity to consciously perceive the phenomenon. It may be that the effect of ASMR reaches everyone but remains unconscious for most of the population. Furthermore, from the comparison between pupil diameter during ASMR videos and Arousal (Low and High) videos, our results showed a significant difference (Low Arousal = 3.89 mm; High Arousal = 3.79 mm), demonstrating that ASMR pupil dilation is the largest.

Regarding the measurement of arousal, today, thanks to new technologies, arousal can be investigated by using different tools: first, the detection of skin conductance (biofeedback). This index is very sensitive to minimal fluctuations in arousal, measured without any time difference. Another measurement method often used is the detection of the heart rate [40]. By these detection methods, the authors claimed that an elevated arousal manifests itself with an accelerated heart rate and high skin conductance. Likewise, pupillary dilation also reflects elevated arousal [21]. Regarding the studies related to arousal, measured through the use of the electroencephalogram (EEG), increases in the frontal-right Theta frequencies and in the parietal Beta frequencies can be noticed (in moments of high perceived arousal), together with specific changes in the valence in the temporal Beta [41].

Another point of view we considered to examine the ASMR phenomenon was the brain activity. A lot of research has been reported; for example, Fredborg et al. [12] and Lee et al. [13] found an increased Alpha wave activity, associated with a certain form of meditative state, and Smith et al. [8,9] observed a greater brain connectivity in the areas involved with the Default Mode Network (DMN). The DMN [42] is a brain network that comprises the hippocampus, medial prefrontal cortex, temporal lateral and temporal-parietal regions, and posterior medial cortex [43,44]: these brain areas are activated by states of resting, spontaneous mental activity, spontaneous thinking, self-referenced thoughts, daydreaming imagination, plans, and expectations. Andreasen et al. [45] noted that the resting state “consists of a mixture of freely wandering past recollection, future plans, and other personal thoughts and experiences.” These regions are central components of the core brain system that is consistently activated in humans during undirected mental states. From our NeuroSky brain waves track, comparing ASMR and other videos, it is clear that ASMR does not differ from the Rest video. We believe that the lack of differences in brain activity between ASMR and Rest is caused by this brain activity. In brief, according to Smith et al. [8,9] and Fredborg et al. [12], it could be that ASMR could trigger the brain’s DMN status and elicit a sort of meditative state. The usage of NeuroSky technology is, of course, a “double-edged sword”: on the one hand, it is very robust to motion artifacts, so it allows ecological data mining; on the other hand, its sensitivity and precision are very low. Such limitations, of course, make it not necessarily meaningful, nor correct, to directly compare the NeuroSky output values with other EEG systems. It is also conceivable that NeuroSky could not be sensitive enough to recognize the EEG frequency differences between Rest and ASMR conditions, because they could be too subtle.

As a lot of research has supported, ASMR could be a complex emotional experience that comprises feelings of both relaxation and excitement; paradoxical physiological responses to ASMR showed decreased heart rate and increased skin conductance levels [2]. It was also demonstrated by Koo et al. [15] that ASMR is strictly related to arousal. From our results, we detected for the first time in the literature a discrepancy between brain arousal and pupil diameter, apparently specific to ASMR videos (similar to the dyscrasia found by Poerio et al. [2]). At the same time, we noted some features usually associated with an increase in arousal, demonstrated by the increase in pupil diameter (Table 5), and at the same time, some features usually coupled with a decrease in arousal were demonstrated with data collected by NeuroSky (Table 6). In line with Poerio et al. [2], we hypothesized a possible causal effect of such dyscrasia on the ASMR-related perceptual complex phenomenology. The data presented so far showed that ASMR appears as a complex phenomenon characterized by paradoxical manifestations. Until today, several studies have assumed the existence of this idiosyncrasy, but there is no clear explanation for this yet. In our opinion, in all the individuals, in front of an ASMR video, the DMN is activated and the pupillary diameter is increased, but some subjects, who claimed to perceive “Many” tingles, tended to resist these idiosyncratic manifestations (probably because of the activation of the ASMR). A possible explanation could rely on a struggle of cognitive systems in restoring balance, homeostasis, so that tingles could be the manifestation of this idiosyncrasy/mechanism. People who felt more tingles probably tended to resist flooding, and probably did not want to let loose; this could be justified both by the results of IRI-Empathic Concern (higher) and by those of the Telepresence Scale (lower).

Similarly, also with the results derived from ASMR 15 [35], it is noted that the Relaxation subscale is not related to the NeuroSky brain waves; this could mean that the relaxation resulting from the ASMR is not related to changes in brain frequencies. This does not mean that relaxation does not occur during ASMR, but it is probably linked to other aspects, still not explored. In our view, this mental state could represent a sort of paradoxical desynchronization. Obviously, all that has been said above represents a starting point that needs more research to deepen the idea of paradoxical desynchronization and to understand its relationship with the perceptive phenomenology of ASMR. It should be clarified that, although the average amount of tingles was very low, the difference between “Few” and “Many” tingles was significant, and the amount of tingles detected is more or less in line with the literature. Furthermore, the low amount of tingles could certainly be due to the laboratory conditions, which probably reduced immersion and relaxation. Nevertheless, this could be a limitation, and these data are extremely interesting and we believe that further research is necessary.

In conclusion, it is clear that people use ASMR videos to relax, induce sleep, and reduce anxiety, but despite the relaxing state in which people affirm to be, physiological data also suggest an activation component. Our data can be a starting point to understanding the general framework regarding the mechanisms underlying the ASMR; because of the complex nature of the phenomenon, it is necessary to understand how this activation is consciously perceived by individuals, and also, how, and when, this idiosyncrasy occurs.

## Figures and Tables

**Table 1:** Descriptive statistics of the psychological variables. The telepresence measure refers to the experimental experience.

Variable	Mean	*SD*	Skewness	Kurtosis
Empathic concern (IRI)	27.1	4.5	*−*0.61	0.54
Personal distress (IRI)	19.1	5.4	0.11	*−*0.49
Telepresence	18.5	5.0	*−*0.30	*−*0.90

**Table 2:** Descriptive statistics of the pupil variables for each video. In the table, we report both the original and adjusted values for skewness and kurtosis [37].

Variable	Mean	*SD*	Skewness	Adj. Skew.	Kurtosis	Adj. Kurt.
Pupil diameter: Rest	3.39	1.23	*−*0.14	−0.14	1.41	1.45
Pupil diameter: High arousal	3.79	1.21	*−*0.59	−0.61	0.45	0.46
Pupil diameter: Low arousal	3.89	1.33	*−*0.59	−0.61	0.41	0.42
Pupil diameter: Control	3.96	1.55	*−*0.18	−0.18	0.30	0.31
Pupil diameter: ASMR	4.32	1.31	*−*0.46	−0.47	0.16	0.16

**Table 3:** Descriptive statistics of the neurophysiological variables during ASMR video. Brain wave values are measured in ASIC-EEG-POWER.

NeuroSky Frequency Bands	Mean	*SD*	Skewness	Kurtosis
Delta	305,851.3	198,037.4	1.32	2.41
Theta	70,877.6	39,115.7	2.40	3.11
Low Alpha	17,854.7	7942.3	1.61	3.01
High Alpha	17,779.1	6857.2	0.93	2.13
Low Beta	13,803.4	5560.5	1.32	3.81
High Beta	14,096.5	9585.7	2.00	4.78
Low Gamma	5805.3	4401.6	1.68	2.33
Mid Gamma	2826.2	2173.0	2.38	6.59

**Table 4:** Descriptive statistics of the ASMR variables (ASMR-Experience and ASMR 15 dimensions).

Variable	Mean	*SD*	Skewness	Kurtosis
ASMR-Experience	1.97	1.29	1.33	0.95
ASMR 15 Altered Consciousness	8.13	3.94	0.57	*−*0.67
ASMR 15 Sensation	11.64	5.57	0.22	*−*1.36
ASMR 15 Relaxation	7.57	3.48	0.29	*−*1.05
ASMR 15 Affect	7.42	3.35	0.41	*−*0.70

**Table 5:** Dependent sample *t*-test analysis of pupil diameter between ASMR and other conditions.

Conditions	Average Pupil Diameter	Difference	S.E.	Student’s *t*
Rest	3.39	−0.92	0.14	−6.50 ***
ASMR	4.32			
High-Arousal	3.79	−0.53	0.13	−4.19 ***
ASMR	4.32			
Low-Arousal	3.89	−0.43	0.15	−2.76 ***
ASMR	4.32			
Control	3.96	−0.36	0.14	−2.60 ***
ASMR	4.32			

***: *p* < 0.001; Note. Pupil diameter is expressed in mm. ASMR = Autonomous Sensory Meridian Response.

**Table 6:** Comparison of NeuroSky frequency bands between ASMR and other conditions, with the dependent sample Student’s *t*-test. T sign represents the direction of the difference; in this case, positive sign means higher values under the ASMR condition.

NeuroSky Frequency Bands	Rest	High Arousal	Low Arousal	Control
Delta	−0.34	−2.12 *	−2.61 **	−2.94 **
Theta	−0.46	−0.58	−2.15 *	−2.66
Low Alpha	−0.30	−0.70	−1.92 *	−1.52
High Alpha	0.07	−1.21	−3.49 ***	−2.56 **
Low Beta	−0.33	−2.64 **	−2.38 *	−2.02 *
High Beta	−0.04	0.31	−1.42	−0.41
Low Gamma	−1.55	−0.12	−1.31 *	−2.89 **
Mid Gamma	−0.70	−0.98	−1.45	−2.19 **

*: *p* < 0.05; **: *p* < 0.01; ***: *p* < 0.001.

**Table 7:** Differences between receivers of higher vs. lower tingles in IRI and Telepresence Scale.

Measures	Sample	N	Mean (*SD*)	Adj. Skewness	Adj. Kurtosis	Student’s *t*
IRI Empathic concern	Few	17	25.35 (5.33)	−0.74	0.85	2.34 *
	Many	20	28.70 (3.28)	0.16	−0.63	
IRI Personal distress	Few	17	22.88 (4.82)	0.10	−1.11	−2.27 *
	Many	20	19.55 (4.12)	−0.15	−0.18	
Telepresence	Few	17	18.24 (4.45)	0.19	−1.31	−2.03 *
	Many	20	15.00 (5.14)	−0.45	−0.23	

* = *p* < 0.05; Note. We reported adjusted values of Skewness and Kurtosis for the variables considered in the two subsamples. Values appear as always within an acceptable range (i.e., around “−1” and “+1”).

**Table 8:** Correlations between ASMR-15 and NeuroSky frequency bands.

NeuroSky Frequency Bands	Altered Consciousness	Sensations	Relaxation	Affect
Delta	0.05	0.29 **	−0.06	0.15
Theta	0.10	0.33 ***	0.01	0.18
Low Alpha	0.05	0.19	0.02	0.17
High Alpha	0.02	0.19	0.06	0.15
Low Beta	0.08	0.30 ***	0.05	0.20 *
High Beta	0.18	0.37 ***	0.10	0.23 *
Low Gamma	0.22	0.37 ***	0.05	0.21 *
Mid Gamma	0.27	0.38 ***	0.03	0.21 *
Average	0.06	0.31 ***	−0.04	0.17

*: *p* < 0.05; **: *p* < 0.01; ***: *p* < 0.001.

## Data Availability

The data that support the findings of this study are available from the corresponding author, [CP].

## References

[1] Barratt E.L., Davis N.J. (2015). Autonomous sensory meridian response (asmr): A flow-like mental state. PeerJ.

[2] Poerio G.L., Blakey E., Hostler T.J., Veltri T. (2018). More than a feeling: Autonomous sensory meridian response (asmr) is characterized by reliable changes in affect and physiology. PLoS ONE.

[3] Ohta Y., Inagaki K. Evaluation of the Effect of ASMR on Reduction of Mental Stress: EEG Study. Proceedings of the 2021 IEEE 3rd Global Conference on Life Sciences and Technologies (LifeTech).

[4] Andersen J. (2015). Now you’ve got the shiveries: Affect, intimacy, and the asmr whisper community. Telev. New Media.

[5] Morales R., Ramírez-Benavides D., Villena-Gonzalez M. (2021). Autonomous Sensory Meridian Response self-reporters showed higher scores for cognitive reappraisal as an emotion regulation strategy. PeerJ.

[6] McErlean AB J., Banissy M.J. (2017). Assessing individual variation in personality and empathy traits in self-reported autonomous sensory meridian response. Multisens. Res..

[7] Klausen H.B. (2019). Safe and sound. SoundEffects Interdiscip. J. Sound Sound Exp..

[8] Smith S.D., Fredborg B., Kornelsen J. (2017). An examination of the default mode network in individuals with autonomous sensory meridian response (asmr). Soc. Neurosci..

[9] Smith S.D., Fredborg B.K., Kornelsen J. (2020). Functional connectivity associated with five different categories of autonomous sensory meridian response (asmr) triggers. Conscious. Cogn..

[10] Lochte B.C., Guillory S.A., Richard C.A., Kelley W.M. (2018). An fmri investigation of the neural correlates underlying the autonomous sensory meridian response (asmr). BioImpacts BI.

[11] Wang X., Yang X., Sun Y., Su Y. (2020). The influence of autonomous sensory meridian response on individual’s executive function. Q. J. Exp. Psychol..

[12] Fredborg B.K., Champagne-Jorgensen K., Desroches A.S., Smith S.D. (2021). An electroencephalographic examination of the autonomous sensory meridian response (asmr). Conscious. Cogn..

[13] Lee D.J., Kulubya E., Goldin P., Goodarzi A., Girgis F. (2018). Review of the neural oscillations underlying meditation. Front. Neurosci..

[14] Seifzadeh S., Moghimi E., Torkamani F., Ahsant N. (2021). Cortical Activation Changes Associated with Autonomous Sensory Meridian Response (ASMR): Initial Case Report. Front. Biomed. Technol..

[15] Koo B., Kim D.H., Ha J., Kim L. An approach for assessing arousal characteristics of ASMR using electroencephalographic power. Proceedings of the 2021 9th International Winter Conference on Brain-Computer Interface (BCI).

[16] Geva R., Zivan M., Warsha A., Olchik D. (2013). Alerting, orienting or executive attention networks: Differential patterns of pupil dilations. Front. Behav. Neurosci..

[17] Bradshaw J. (1967). Pupil size as a measure of arousal during information processing. Nature.

[18] Dos Santos R.D.O.J., de Oliveira J.H.C., Rocha J.B., Giraldi J.D.M.E. (2015). Eye tracking in neuromarketing: A research agenda for marketing studies. Int. J. Psychol. Stud..

[19] Sáiz-Manzanares M.C., Rodríguez-Díez J.J., Marticorena R., Zaparaín M.J., Cerezo R. (2020). Lifelong Learning from Sustainable Education: An Analysis with Eye Tracking and Data Mining Techniques. Sustainability.

[20] Valtakari N.V., Hooge I.T., Benjamins J.S., Keizer A. (2019). An eye-tracking approach to autonomous sensory meridian response (asmr): The physiology and nature of tingles in relation to the pupil. PLoS ONE.

[21] Bradley M.M., Miccoli L., Escrig M.A., Lang P.J. (2008). The pupil as a measure of emotional arousal and autonomic activation. Psychophysiology.

[22] Libby W.L., Lacey B.C., Lacey J.I. (1973). Pupillary and cardiac activity during visual attention. Psychophysiology.

[23] Steinhauer S.R., Siegle G.J., Condray R., Pless M. (2004). Sympathetic and parasympathetic innervation of pupillary dilation during sustained processing. Int. J. Psychophysiol..

[24] (2020). Mindwave mobile 2: User Guide [Computer Software Manual]. Retrieved from NeuroSky, I. https://store.neurosky.com/pages/mindwave.

[25] NeuroSky, I. (n.d.). Eeg Band Power Values: Units, Amplitudes, and Meaning [Computer Software Manual]. http://support.neurosky.com/kb/development-2/eeg-band-power-values-units-amplitudes-and-meaning.

[26] Pop-Jordanova N., Pop-Jordanov J. (2005). Spectrum-weighted EEG frequency (“brain-rate”) as a quantitative indicator of mental arousal. Prilozi.

[27] Heraz A., Frasson C. (2011). Towards a brain-sensitive intelligent tutoring system: Detecting emotions from brainwaves. Adv. Artif. Intell..

[28] PupilCore Guide (2019). https://pupil-labs.com/products/core/.

[29] Cash D.K., Heisick L.L., Papesh M.H. (2018). Expectancy effects in the autonomous sensory meridian response. PeerJ.

[30] Li B.J., Bailenson J.N., Pines A., Greenleaf W.J., Williams L.M. (2017). A public database of immersive vr videos with corresponding ratings of arousal, valence, and correlations between head movements and self report measures. Front. Psychol..

[31] Owens D., Leibowitz H. (1975). The fixation point as a stimulus for accommodation. Vis. Res..

[32] Thaler L., Schütz A.C., Goodale M.A., Gegenfurtner K.R. (2013). What is the best fixation target? the effect of target shape on stability of fixational eye movements. Vis. Res..

[33] Davis M.H. (1980). Interpersonal Reactivity Index.

[34] Kim T., Biocca F. (1997). Telepresence via television: Two dimensions of telepresence may have different connections to memory and persuasion. J. Comput. Commun..

[35] Roberts N., Beath A., Boag S. (2019). Autonomous sensory meridian response: Scale development and personality correlates. Psychol. Conscious. Theory Res. Pract..

[36] IBM Corp (2020). IBM SPSS Statistics for Macintosh, Version 27.0.

[37] Pham H. (2006). Springer Handbook of Engineering Statistics.

[38] Keizer A., Chang T.H., O’Mahony C.J., Schaap N.S., Stone K.D. (2020). Individuals who experience autonomous sensory meridian response have higher levels of sensory suggestibility. Perception.

[39] Bjelić T. (2016). Digital care. Women Perform. A J. Fem. Theory.

[40] Taylor S.P., Epstein S. (1967). The measurement of autonomic arousal: Some basic issues illustrated by the covariation of heart rate and skin conductance. Psychoso Matic Med..

[41] Stenberg G. (1992). Personality and the eeg: Arousal and emotional arousability. Personal. Individ. Differ..

[42] Shulman G.L., Corbetta M., Fiez J.A., Buckner R.L., Miezin F.M., Raichle M.E., Petersen S.E. (1997). Searching for activations that generalize over tasks. Hum. Brain Mapp..

[43] Buckner R.L., Andrews-Hanna J.R., Schacter D.L., Kingstone A., Miller M.B. (2008). The brain’s default network: Anatomy, function, and relevance to disease. The Year in Cognitive Neuroscience.

[44] McVay J.C., Kane M.J. (2009). Conducting the train of thought: Working memory capacity, goal neglect, and mind wandering in an executive-control task. J. Exp. Psychol. Learn. Mem. Cogn..

[45] Andreasen N.C., O’Leary D.S., Cizadlo T., Arndt S., Rezai K., Watkins G.L., Hichwa R.D. (1995). Remembering the past: Two facets of episodic memory explored with positron emission tomography. Am. J. Psychiatry.

